# Left Ventricular Unloading During Veno-Arterial ECMO: A Simulation Study

**DOI:** 10.1097/MAT.0000000000000755

**Published:** 2018-03-10

**Authors:** Dirk W. Donker, Daniel Brodie, José P. S. Henriques, Michael Broomé

**Affiliations:** From the * Department of Intensive Care Medicine, University Medical Center Utrecht, Utrecht University, The Netherlands; † Division of Pulmonary, Allergy and Critical Care Medicine, Columbia University College of Physicians and Surgeons/New York-Presbyterian Hospital, New York, New York; ‡ Department of Cardiology, Academic Medical Center, University of Amsterdam, Amsterdam, The Netherlands; § ECMO Department, Karolinska University Hospital, Stockholm, Sweden; ¶ Anaesthesiology and Intensive Care, Department of Physiology and Pharmacology, Karolinska Institute, Stockholm, Sweden; ‖ School of Technology and Health, Royal Institute of Technology, Stockholm, Sweden.

**Keywords:** Veno-arterial extracorporeal membrane oxygenation, extracorporeal life support, cardiogenic shock, cardiovascular modeling, computer simulation

## Abstract

Veno-arterial extracorporeal membrane oxygenation (VA ECMO) is widely used in cardiogenic shock. It provides systemic perfusion, but left ventricular (LV) unloading is suboptimal. Using a closed-loop, real-time computer model of the human cardiovascular system, cardiogenic shock supported by peripheral VA ECMO was simulated, and effects of various adjunct LV unloading interventions were quantified. After VA ECMO initiation (4 L/min) in cardiogenic shock (baseline), hemodynamics improved (increased to 85 mm Hg), while LV overload occurred (10% increase in end-diastolic volume [EDV], and 5 mm Hg increase in pulmonary capillary wedge pressure [PCWP]). Decreasing afterload (65 mm Hg mean arterial pressure) and circulating volume (−800 mL) reduced LV overload (12% decrease in EDV and 37% decrease in PCWP) compared with baseline. Additional intra-aortic balloon pumping only marginally decreased cardiac loading. Instead, adjunct Impella™ enhanced LV unloading (23% decrease in EDV and 41% decrease in PCWP). Alternative interventions, for example, left atrial/ventricular venting, yielded substantial unloading. We conclude that real-time simulations may provide quantitative clinical measures of LV overload, depending on the degree of VA ECMO support and adjunct management. Simulations offer insights into individualized LV unloading interventions in cardiogenic shock supported by VA ECMO as a proof of concept for potential future applications in clinical decision support, which may help to improve individualized patient management in complex cardiovascular disease.

## Abstract 2

Supplemental Digital Content is available in the text.

Veno-arterial extracorporeal membrane oxygenation (VA ECMO) is becoming an established short-term mechanical support modality for patients with severe cardiogenic shock refractory to conventional therapy. Recent advances in technology have greatly facilitated the application of VA ECMO, allowing for the immediate initiation of full circulatory support in a variety of clinical settings.^1^ Yet, individualized management of VA ECMO remains challenging in clinical practice because the optimal balance between left ventricular (LV) unloading and systemic perfusion can be very delicate and depends on the underlying disease and potential for myocardial recovery.^2^ Moreover, the individual hemodynamic state is continuously affected by patient- and treatment-related factors that dynamically change throughout the clinical course. It is increasingly recognized that VA ECMO may cause significant cardiac mechanical overload, that is, increased stress and strain exerted on the LV myocardium.^3,4^ This potentially hampers cardiac recovery, promotes adverse myocardial remodeling, and ultimately causes irreversible heart failure. LV dilatation may ultimately ensue, accompanied by increased filling pressures and aggravation of pulmonary edema.^3,5^ Thereby, gas exchange is impaired in the pulmonary circulation causing deoxygenated blood to be ejected from the LV into the aorta and coronary circulation. As a consequence of insufficient LV unloading and persistent pulmonary edema, a vicious circle of proximal aortic hypoxemia and myocardial ischemia may arise, which in turn impairs LV contractility and recovery. Therefore, it is of utmost importance to create optimal cardiac loading conditions during VA ECMO. Different strategies and a variety of interventions such as intra-aortic balloon pump (IABP), Impella, atrial septostomy, and LV venting have been proposed.^3^ However, the detection of LV overload remains cumbersome, and in clinical practice, it is virtually impossible to predict whether an individual patient would benefit from unloading interventions.^3^ Moreover, it remains elusive to predict the exact degree of LV unloading at the bedside that could be achieved by applying a specific intervention in an individual patient on VA ECMO. Here, we simulate interventions, which have been described in the literature, aimed at unloading the LV during VA ECMO. To understand their practical applicability and to quantify the expected degree of unloading, we simulated cases in a real-time, closed-loop computer model of the human cardiovascular system. This patient-specific approach has recently been demonstrated to generate valid information and could potentially serve as a future clinical decision support tool, which may help to improve individualized patient management in complex cardiovascular disease.^2,6,7^

## Methods

### Real-Time Cardiovascular Simulation Model

Parameters in a cardiovascular computer simulation model were fitted to generic human clinical data from an adult 70 kg individual with severe, predominant LV systolic heart failure, as published before and detailed in the Appendix^2,6–8^ (see Appendix, Supplemental Digital Content, http://links.lww.com/ASAIO/A235). Model outputs are real-time pressures, flows, volumes, and oxygen saturations in the heart and vascular system. The simulated case was supported by VA ECMO alone and in conjunction with various interventions to optimize cardiac loading conditions.

### Simulation of LV Failure and Evaluation of Loading Conditions

**Figure 1.**
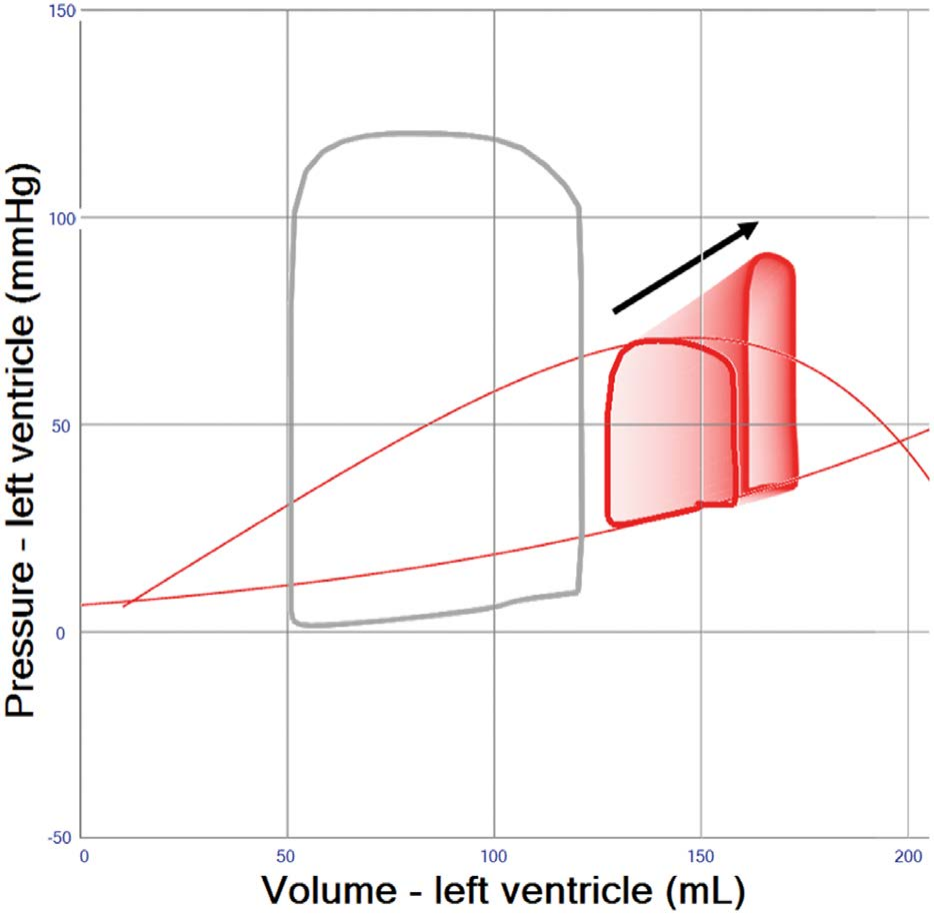
Pressure–volume loop simulation of left ventricular (LV) failure. Normal physiology shown in gray loop for comparison. Red loop shows an LV with reduced systolic contractility, increase in passive LV diastolic stiffness and blood volume (see text) to mimic a clinical case of cardiogenic shock. Red thin lines indicate end-systolic and end-diastolic pressure–volume relations. The black arrow shows an increasing VA extracorporeal membrane oxygenation (ECMO) support 0–4 L/min with resulting decrease in stroke volume and dilatation of the LV.

Severe LV heart failure was simulated by decreasing contractility as detailed in the Appendix (**Figure 1** and **Table 1**). Sinus rhythm was set to a heart rate of 100 bpm. Blood volume was increased from 5600 to 6400 ml to simulate the pathophysiology of heart failure, causing a further increase in LV dilatation and pulmonary capillary wedge pressure (PCWP) to 30 mm Hg. Cardiac index (output) decreased from 3.9 (7.0) to 1.7 (3.1) L/min/m^2^ compatible with cardiogenic shock. LV oxygen consumption was estimated by calculation of the pressure–volume–area (see Appendix Figure A2, Supplemental Digital Content, http://links.lww.com/ASAIO/A235).^9^ A simulated pressure–volume (PV) loop illustrating normal conditions and LV systolic heart failure before treatment is depicted in **Figure 1**, also featuring end-systolic and end-diastolic PV relations in heart failure.

**Table 1.**
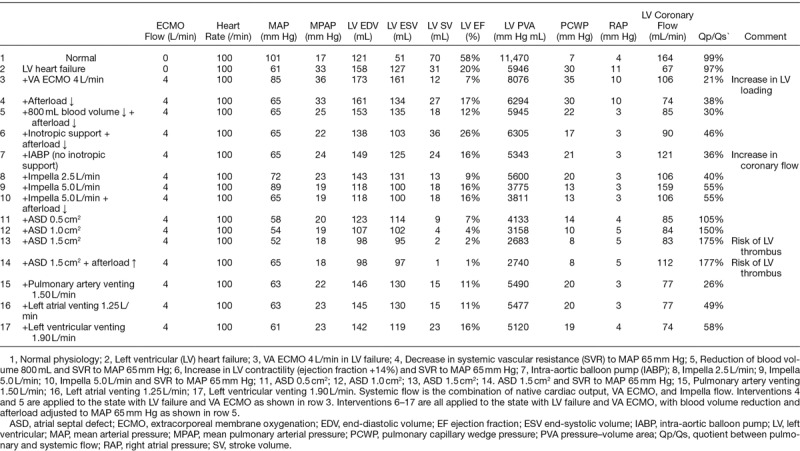
Hemodynamic Data for Normal Physiology, Isolated Left Ventricular (LV) Failure and LV Failure Supported With VA ECMO and Various Adjunct Therapies

### ECMO Simulation

VA ECMO was simulated with a fixed blood flow (0–4 L/min) mimicking a peripheral, bi-femoral cannulation with right atrial venous drainage and retrograde reinfusion in the descending aorta (**Figure 1**). All adjunct LV unloading interventions as incorporated in the simulation, that is IABP, Impella, atrial septal defects, and LV venting are detailed in the Appendix, Supplemental Digital Content, http://links.lww.com/ASAIO/A235.

## Results

### Effects of VA ECMO Support Flow

A gradual increase of VA ECMO flow in the model (blood flow 0 to 4 L/min) results in progressive LV dilatation with a decrease of LV stroke work (**Figure 1** and **Table 1** (Row 3) but an increase in oxygen consumption in accordance with the pressure–volume–area concept (see Appendix, Supplemental Digital Content, http://links.lww.com/ASAIO/A235). The increase in VA ECMO flow is accompanied by an increase in systolic LV pressure and arterial blood pressure (mean arterial pressure [MAP] increase to 85 mm Hg; (**Figure 1** and **Table 1** (Row 3)). LV stroke volumes decrease although the total cardiac output including VA ECMO support flow increases (**Table 1** (Row 3)).

### Pre-/Afterload Reduction and Inotropic Support

**Figure 2.**
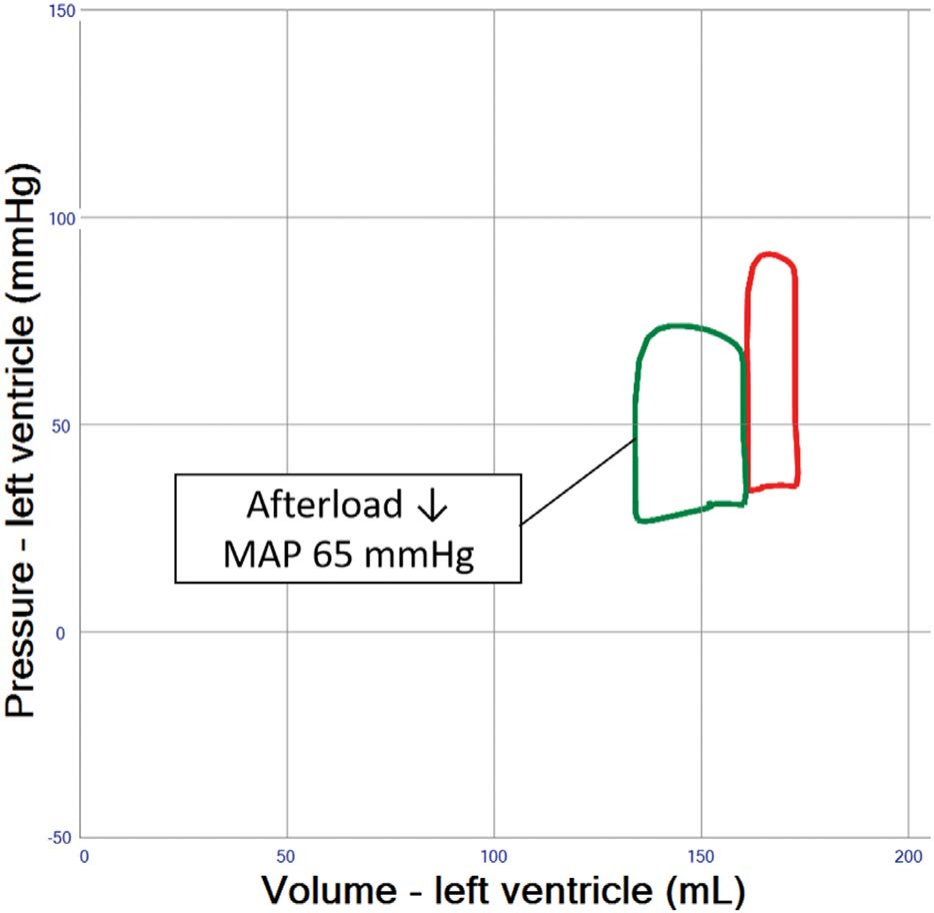
Pressure–volume loop analysis representing the effects of afterload reduction in left ventricular systolic failure supported by veno-arterial extracorporeal membrane oxygenation (VA ECMO) 4 L/min. Afterload (systemic vascular resistance) is reduced until systemic mean arterial blood pressure (MAP) reaches 65 mm Hg, an accepted target in clinical care for patients with cardiogenic shock providing a reasonable compromise between low afterload, systemic and coronary perfusion pressure.

**Figure 3.**
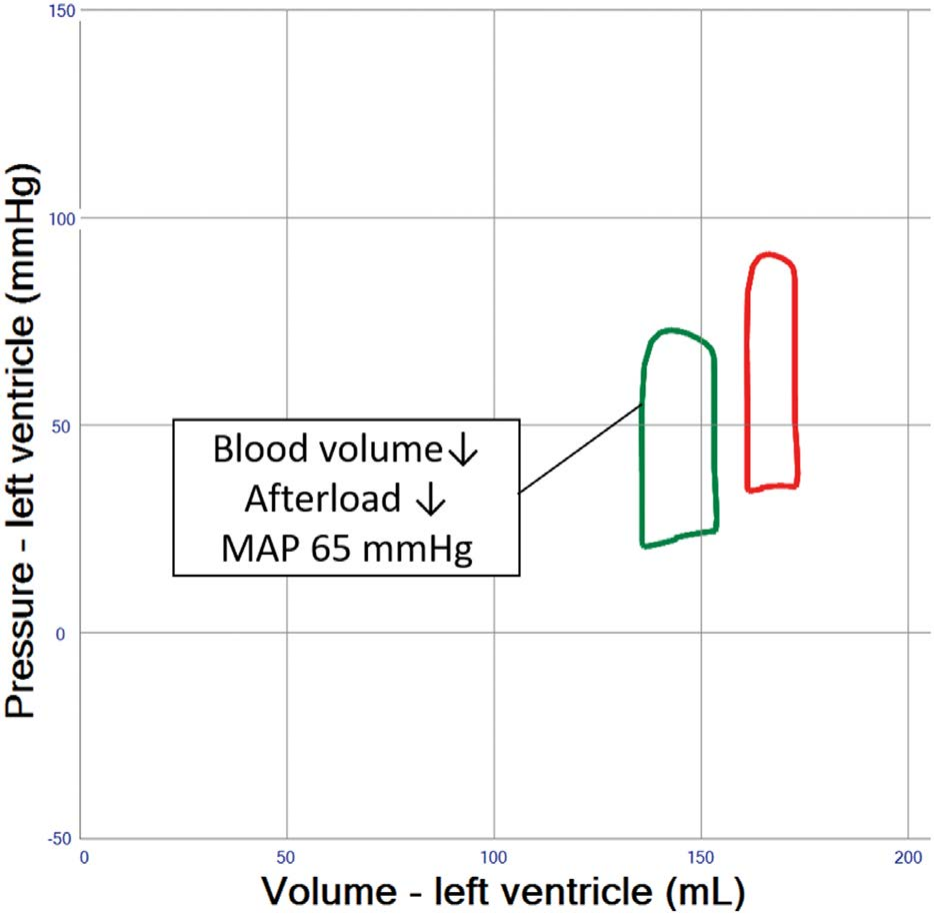
Pressure–volume loop analysis of both afterload and blood volume reduction in left ventricular (LV) systolic failure supported by veno-arterial extracorporeal membrane oxygenation (VA ECMO) 4 L/min. Blood volume is reduced by 800 mL reaching a normal blood volume. Afterload (systemic vascular resistance) is then reduced until mean arterial blood pressure (MAP) reaches 65 mm Hg, as shown in Figure 2. Stroke volume and end-diastolic volume is reduced as compared with afterload reduction alone, thereby illustrating the importance of blood volume reduction in LV unloading.

**Figure 4.**
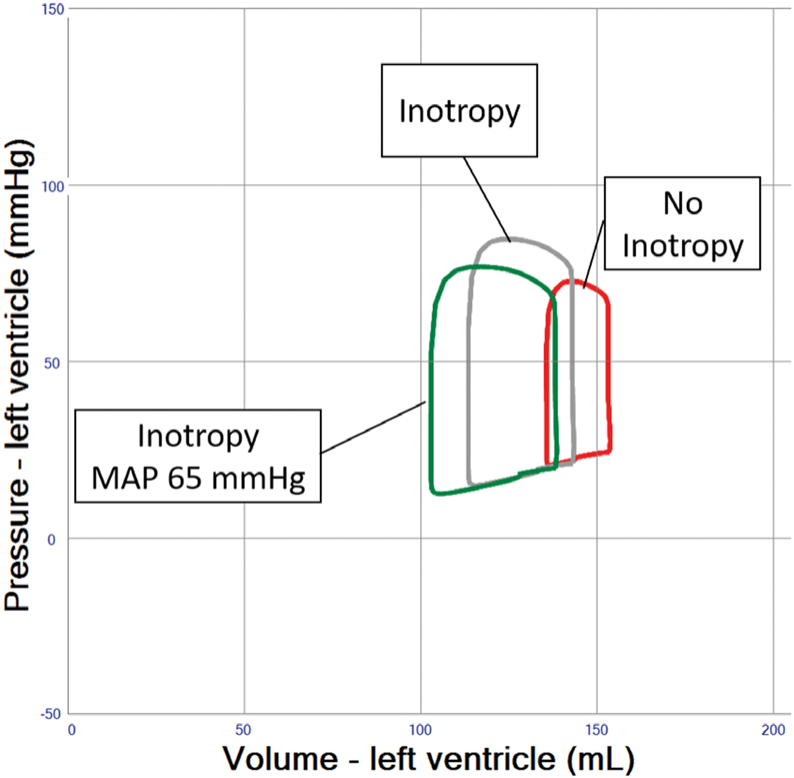
Pressure–volume loop analysis of inotropic drug effects in left ventricular failure and veno-arterial extracorporeal membrane oxygenation (VA ECMO) 4 L/min. Inotropy only (gray loop) reduces end-diastolic volume and increases stroke volume as well as systemic mean arterial blood pressure (MAP) as compared with no inotropy (red loop). Systemic vascular resistance is finally readjusted down to an MAP of 65 mm Hg, providing further unloading (green loop).

The rise in MAP observed after increasing VA ECMO blood flow rates (**Figure 1** and **Table 1** (Row 3)) is associated with an increase in afterload. However, this increase in afterload may be mitigated by striving for a minimally acceptable MAP of 65 mm Hg. This may be achieved by using ino-dilators, a reduction of vasopressor treatment or occasionally the addition of venous or arterial vasodilators, which in turn may create more favorable myocardial loading conditions (**Figure 2**). The effect of vasodilatation on LV loading is, however, limited and usually not sufficient to reduce LV end-diastolic volume (EDV) by more than 10% (**Figure 2** and **Table 1** (Row 4)). A reduction in blood volume of 500–1000 mL will decrease right ventricular filling, pulmonary blood flow, and LV filling pressures, yet the resultant reduction of LV EDV is limited to 5 to 10 mL (**Figure 3** and **Table 1** (Row 5)). Inotropic therapy increased LV ejection fraction in the model by 10% and reduced end-diastolic volume by 10 mL, while stroke volume as well as systemic MAP increased (**Figure 4**). When systemic vascular resistance is finally readjusted down to an MAP of 65 mm Hg, further LV unloading (ejection fraction +14%, end-diastolic volume −15 mL) is achieved (**Figure 4** and **Table 1**(Row 6)).

### Intra-Aortic Balloon Pump Combined With VA ECMO

**Figure 5.**
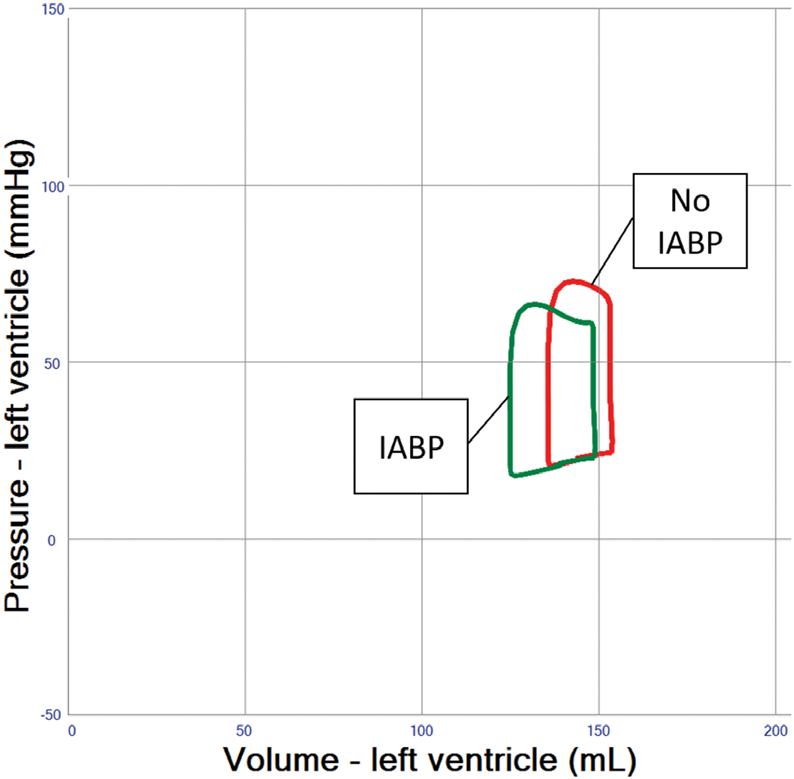
Pressure–volume loop analysis of intra-aortic balloon pumping (IABP) effects in left ventricular failure and veno-arterial extracorporeal membrane oxygenation (VA ECMO) 4 L/min. End-diastolic volume decreases only minimally and stroke volume increases as a result of decreasing afterload. In addition, diastolic systemic blood pressure increases providing improved coronary blood flow (Table 1, not shown in pressure–volume loop).

The simulation demonstrates an increase in pulsatility and LV stroke volume by 5% to 10% because of a reduction of afterload when an IABP is used in severe heart failure patients treated with VA ECMO, yet PCWP and LV EDV remain practically unchanged (**Figure 5** and **Table 1** (Row 7)), whereas coronary blood flow is improved (**Table 1** (Row 7)).

### Impella Combined With VA ECMO

**Figure 6.**
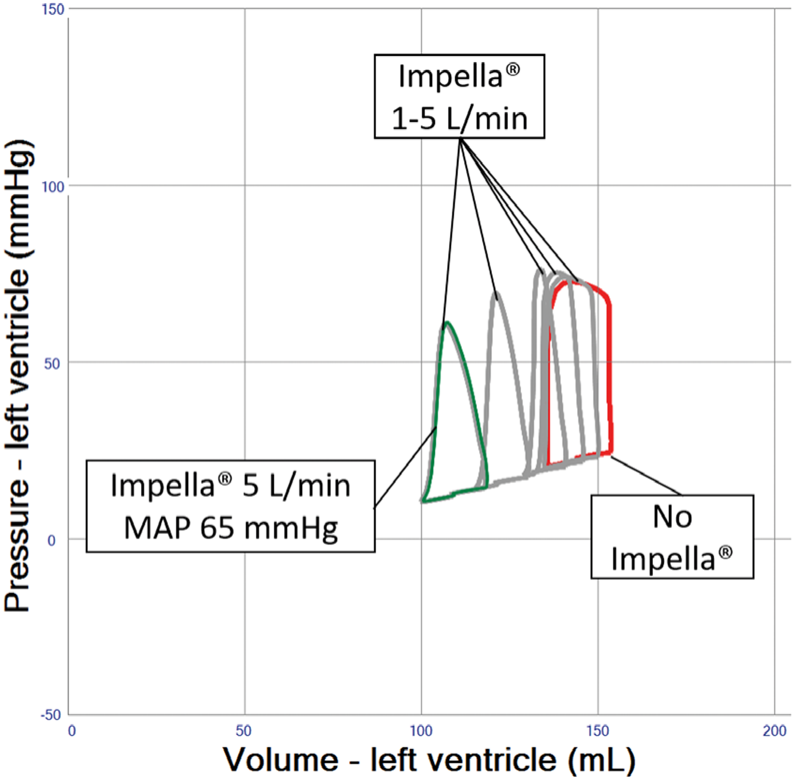
Pressure–volume loop analysis of Impella effects in left ventricular systolic failure and veno-arterial extracorporeal membrane oxygenation (VA ECMO) 4 L/min. Device flows of 1–5 L/min are shown in gray loops. Systemic vascular resistance is finally readjusted down to systemic mean arterial blood pressure (MAP) 65 mm Hg, resulting in further unloading as indicated in the green loop.

The simulation shows significant LV unloading when using a temporary ventricular support device, the Impella, to support flow during VA ECMO (**Figure 6** and **Table 1** (Row 8–10)). In comparison to the combined IABP/VA ECMO approach, the addition of the Impella devices solely provides continuous blood flow from the LV into the ascending aorta, but neither facilitates physiological aortic valve opening, nor selectively improves diastolic coronary perfusion pressure as seen with the IABP (**Table 1** (Row 8–10)). The stroke volumes seen in the simulation of the Impella device are actually ejected through the device despite a closed aortic valve. Aortic regurgitation is a potential complication of Impella but was not simulated in this generic patient.

### Indirect and Direct LV Venting During VA ECMO

**Figure 7.**
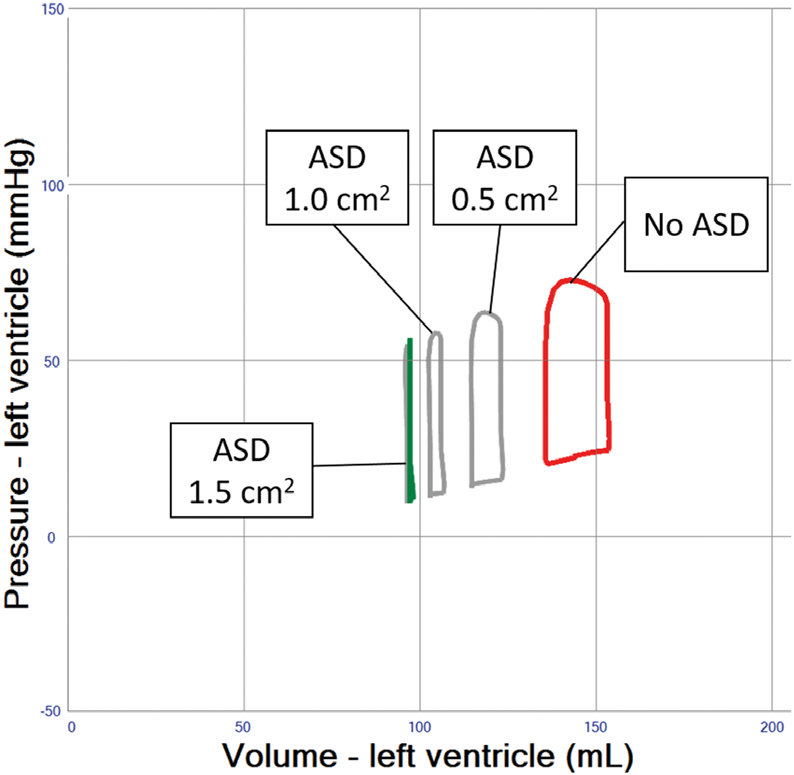
Pressure–volume loop analysis of atrial septostomy in left ventricular (LV) systolic failure and veno-arterial extracorporeal membrane oxygenation (VA ECMO) 4 L/min. The LV is efficiently unloaded with atrial septal defect (ASD) sizes of 0.5, 1, and 1.5 cm^2^. A nonejecting LV is created with the largest defect.

**Figure 8.**
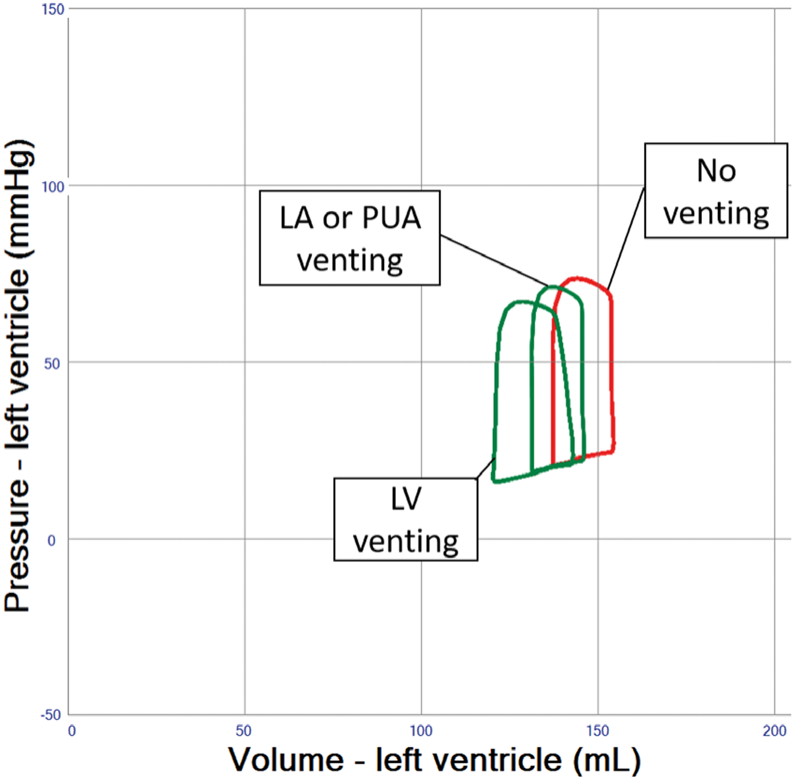
Pressure–volume loop analysis of adjunct venting interventions in left ventricular (LV) systolic failure supported by VA ECMO 4 L/min. Left atrial (LA) and pulmonary artery (PUA) venting show similar hemodynamic effects on the LV pressure–volume loop. LV venting provides more efficient unloading. Flows are determined by actual pressure gradients and resistance of a 3/8″ tubing of 2-m length. Venting flows are 1.3 L/min (LA), 1.5 L/min (PUA) and 1.9 L/min (LV).

**Figure 9.**
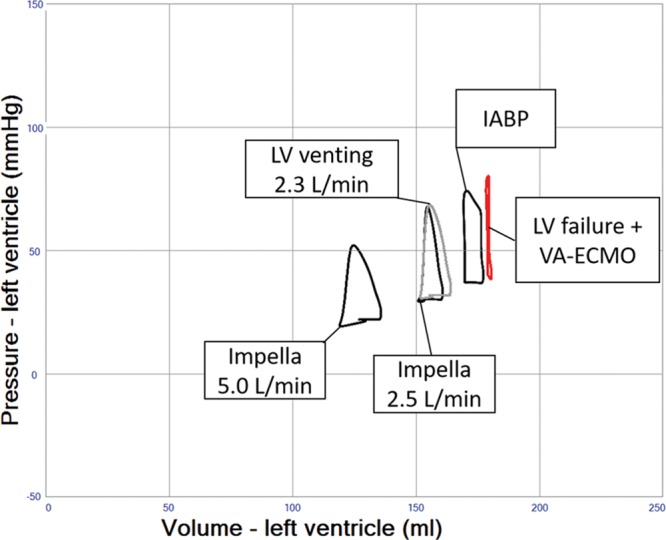
Pressure–volume loop analysis of intra-aortic balloon pump (IABP), left ventricular (LV) venting, and Impella in LV systolic failure and VA ECMO 4 L/min. In this simulation, LV systolic function has further deteriorated (contractility 0.3 mm Hg/mL) to create a clinical state with a nonejecting LV, adjunct therapies all facilitate emptying of the LV.

Indirect LV venting during VA ECMO via atrial septostomy yielded an immediate and substantial LV unloading effect, but sizing of the defect can be critical because too much unloading may result in a nonejecting LV (**Figure 7** and **Table 1** (Row 11–14)). Indirect pulmonary artery venting, or direct transaortic LV venting, as well as surgical LV and LA venting during VA ECMO all resulted in clinically relevant LV unloading effects. (**Figures 8 and 9** and **Table 1** (Row 15–17)).

### Supplemental Material

Animated simulations of cardiogenic shock, VA ECMO and unloading interventions, and related tutorials can be found in the Supplemental Videos 1–12 (see Video 1, Supplemental Digital Content, http://links.lww.com/ASAIO/A236; see Video 2, Supplemental Digital Content, http://links.lww.com/ASAIO/A237; see Video 3, Supplemental Digital Content, http://links.lww.com/ASAIO/A238; see Video 4, Supplemental Digital Content, http://links.lww.com/ASAIO/A239; see Video 5, Supplemental Digital Content, http://links.lww.com/ASAIO/A240; see Video 6, Supplemental Digital Content, http://links.lww.com/ASAIO/A241; see Video 7, Supplemental Digital Content, http://links.lww.com/ASAIO/A242; see Video 8, Supplemental Digital Content, http://links.lww.com/ASAIO/A243; see Video 9, Supplemental Digital Content, http://links.lww.com/ASAIO/A244; see Video 10, Supplemental Digital Content, http://links.lww.com/ASAIO/A245; see Video 11, Supplemental Digital Content, http://links.lww.com/ASAIO/A246; see Video 12, Supplemental Digital Content, http://links.lww.com/ASAIO/A247;).

## Discussion

Our simulation study demonstrates that LV filling pressures, cavity volumes, and myocardial oxygen consumption increase progressively with VA ECMO flow, as reported previously.^2^ The degree of LV loading and unloading during VA ECMO is largely dependent on the absolute VA ECMO flow, the intrinsic LV contractility, or recruitable contractile reserve. Next, it is significantly influenced by specific cardiac unloading measures applied in conjunction with VA ECMO, ranging from medical to percutaneous or surgical interventions.

### Individualized Management of Cardiac Overload in VA ECMO

The bedside integration of hemodynamics, cardiac geometry, and function during VA ECMO is not straightforward. Computer simulation of cardiovascular dynamics using clinical data as input may help to monitor cardiac loading in relation to the degree of VA ECMO support over time.^2^ This approach awaits further clinical validation but has the potential to provide bedside decision support to tailor individualized LV unloading. To the best of our knowledge, the modeling results presented here are unique, as they allow instantaneous quantification of cardiac loading or unloading in a generic patient with severe left heart failure by simulating clinically relevant adjuncts to VA ECMO. Three aspects essentially dictate the degree of individually required LV unloading: avoiding a nonejecting LV, preventing pulmonary edema, and ultimately facilitating optimal LV unloading and myocardial recovery.

The simulations presented here support the relevance of optimal medical management, as fluid removal while minimizing VA ECMO flow, reducing blood pressure, and eventually adding inotropes will significantly reduce PCWP and prevent pulmonary edema (**Figures 2–4** and **Table 1** (Row 4–6). Interventions such as the combined approach of VA ECMO and IABP have long been clinically applied to augment pulsatility, decrease afterload, and improve blood flow in native coronary arteries and bypass grafts.^10,11^ In the simulation, this combined approach showed only limited LV unloading, although pulsatility and increased stroke volume were noted. Recent clinical data support this notion for different clinical settings and do not advocate a routine combination of VA ECMO and IABP.^12^ Clinical studies have shown a slight reduction in PCWP, LV dimensions, and pulmonary edema in line with our simulation.^13,14^

Patients showing PCWP above 25 mm Hg or a virtually nonejecting LV will require interventional or surgical adjunct measures, which theoretically reduce PCWP by more than 5 mm Hg (**Figures 6–9** and **Table 1** (Row 7–17)).

The Impella 2.5, the larger CP, and the 5.0 surgical device have been used in conjunction with VA ECMO and allow clinically relevant cardiac unloading by reduction of right atrial and PCWP, as well as left-sided volumes and pulmonary edema.^15–18^ Our results support the considerable LV unloading potential as a function of Impella flow (**Figure 6** and **Table 1** (Row 8–10)).

The creation of an atrial septal defect is another valid intervention in this setting, as the simulation reveals that LV unloading is immediate and substantial, which has also been verified clinically^19^; but sizing of the defect can be critical because too much unloading may result in a nonejecting LV (**Figure 7** and **Table 1** (Row 13–14)). A well-controlled size of the atrial septal defect can be created with a specially designed percutaneous device available in different sizes and allowing permanent closure after use.^20,21^ Likewise, LV venting via atrial trans-septal cannulation has been reported,^22^ while the hemodynamic effects of percutaneous venting using a cannula positioned in the left atrium are similar to an atrial septostomy, as simulated (**Figures 7 and 8**). Alternatively, percutaneous LV venting by a transaortic catheter via axillary^23^ or femoral artery access^24^ or using a transpulmonary artery catheter has been proposed in clinical reports.^22^ Direct LV venting via an apical access or a cannula vent in the right superior pulmonary vein usually requires sternotomy or thoracotomy.^20,25^ These surgical approaches generally allow larger cannulae, higher flows, and substantial LV unloading (**Figures 8 and 9** and **Table 1** (Row 15–17)), yet carry inherent surgical risks.

The choice for IABP, specific Impella, or direct or indirect percutaneous or surgical LV venting depends on the individual clinical setting. The risks of LV overload, pulmonary edema, and thrombus formation because of a nonejecting LV should be weighed against time expected for recovery and interventional risks. Our analysis demonstrates that every measure taken to adjust LV loading conditions can potentially be scrutinized in advance with an adequate patient-specific simulation. In this way, management of peripheral VA ECMO may potentially be optimized with a minimum of unwanted side effects.

### Limitations

Complex regulatory systems, for example, baroreceptor reflex and other autoregulatory adaptations to hemodynamic changes, have not been simulated to allow a pure analysis of cardiac unloading effects during VA ECMO support. It can be expected that the increase in total cardiac output caused by VA ECMO results in decreased sympathetic activity and may explain minor differences between simulation results and reported clinical and experimental data.^2,4^ Moreover, an increase in heart rate usually accompanying inotropics or enhanced neurohumoral tone cannot be seen in the LV PV loop of a single cardiac cycle, but may affect myocardial oxygen balance unfavorably. Next, although included in the model, mechanical ventilatory settings, related intrathoracic pressure variations, as well as pulmonary shunting and edema have not been simulated to allow an unbiased analysis of LV unloading.^2^ Furthermore, the model does not allow to simulate 3-dimensional blood flow patterns and changes in cardiac geometry, for example, in patients with ischemic heart failure and LV dyssynchrony. However, adding these features would make real-time simulation and interaction with loading conditions impossible when aiming to simulate in the context of a clinically realistic time frame. Similarly, treatment consequences related to hemolysis and coagulation disorders are clinically relevant, but beyond the scope of the current study, while the model allows for changes in hemoglobin/hematocrit, influencing the blood viscosity within a specific simulation.

## Conclusion

Simulation results demonstrate that VA ECMO per se increases LV loading. The combined use of conservative measures may result in acceptable LV unloading in a majority of VA ECMO cases. Adjunct percutaneous or surgical interventions allow substantial LV unloading, which may be justified in well-selected patients. Cardiovascular simulation may in the future potentially be used clinically to improve prediction of hemodynamic and cardiac effects of these interventions to optimize VA ECMO support in individual patients.

## Acknowledgements

M. Broomé constructed the model, performed programming, simulation runs and drafted the manuscript. D. W. Donker, D. Brodie, and J. P. S. Henriques drafted the manuscript. M. Broomé, D. W. Donker, D. Brodie and J. P. S. Henriques all participated in the evaluation of the clinical relevance of the model as being clinically active medical doctors taking care of ECMO patients. All authors read and approved the final manuscript.

## Supplementary Material
