# Supplementary material for: Left Ventricular Unloading During Veno-Arterial ECMO: A Simulation Study
Source: ASAIO J. 2018 Mar 10;65(1):11–20. doi: 10.1097/MAT.0000000000000755 (PMC6325768; doi:10.1097/MAT.0000000000000755)
Supplement: Supplementary file 1 [file mat-65-11-s001.docx]

Left Ventricular Unloading during Veno-Arterial ECMO

A Simulation Study - Appendix

Dirk W. Donker, Daniel Brodie, José P.S. Henriques , Michael Broomé

# Appendix

## Real-time cardiovascular simulation model

**Clinical data were fitted to parameters in a closed-loop, real-time computer simulation model consisting of 27 vascular segments**, 6 in the pulmonary circulation and 21 in the systemic circulation including the coronary circulation**, the four cardiac chambers with corresponding valves, atrial and ventricular septal interactions, the pericardium and intrathoracic pressure, as previously published (Figure A1) ^1-4^.** In short, the cardiac chambers are represented as time-varying elastances and the closed-loop vascular system segments are characterized by non-linear resistances, compliances, inertias and visco-elastances. Valves are opening and closing gradually depending on pressure gradients. No cardiovascular autonomic tone nor auto-regulatory reflexes are included in the simulations. Intrathoracic pressure was set to zero to avoid variability due to circulatory changes during the respiratory cycle.

**Simulation of left ventricular heart failure and evaluation of loading conditions**

Left ventricular (LV) systolic heart failure was simulated by decreasing maximum elastance from 2.8 to 0.5 mmHg/ml, resulting in LV dilation and an increase in LV filling pressures. A further increase in filling pressures was seen when LV diastolic stiffness was increased from 0.05 to 0.08 mmHg/mL to mimic modest diastolic failure. Blood volume was increased from 5600 ml to 6400 ml to simulate the pathophysiology of heart failure, where fluid retention and iatrogenic fluid resuscitation are among the adaptive mechanisms preserving cardiac output, although also causing a further increase in LV dilatation and filling pressures (LV end-diastolic volume 158 mL and pulmonary capillary wedge pressure 30 mmHg). Normally conducted sinus rhythm **was set to a heart rate of 100 bpm. The pericardium was unchanged, thereby constraining cardiac dilatation, as is realistic in acute heart failure. Cardiac output decreased from 7.0 L/min to 3.1 L/min equivalent with a cardiogenic shock state.** LV oxygen consumption was estimated with calculation of the pressure-volume-area (PVA, Figure A2), with the algorithms included in the simulation model. The PVA concept is a well validated measure of cardiac mechanical work incorporating both flow and pressure generation, taking the size of the ventricle and myocardial wall tension into account ^5^. Clinical measurements of PVA requires continuous pressure and volume monitoring of the LV, which may be accomplished with help of a conductance catheter ^6^. If continuous invasive data are not available, it is reasonable to combine peripheral arterial blood pressure with echocardiography measures such as end-diastolic and end-systolic LV diameters as surrogate measures.

**Coronary flow**

Coronary flows are calculated based on a fixed coronary vascular resistance and a driving pressure determined by the difference between the aortic root pressure and mean wall tension. An example of coronary flow in the right and left side of the heart is shown in figure A3.

**Oxygen transport**

The oxygen carrying capacity of blood *C* (*mL* *O2/L blood*) was calculated according to equation A1 ^7^, where *Hb* is the hemoglobin level *(g/L)* and *Sat is the* oxygen saturation *(%)* of the vascular compartment*.* Post-oxygenator saturation (*S_postox_O_2_*) was 100%. Physically dissolved oxygen was not taken into account.

*Equation A1.*

Oxygen saturation was considered homogenous in each compartment and exchange of oxygen between compartments proportional to flow. A uniform hemoglobin level of 11 g/dL was used in all simulations, corresponding to our institutional transfusion limit in adult ECMO patients and expressing mild anemia in critical illness and fluid retention. Total oxygen consumption excluding the heart was set to 250 ml/min (3.6 ml/kg/min). Cardiac oxygen consumption was calculated according to Suga *et al.* depending on the mechanical workload of the heart (in the study between 16 and 22 ml/min). *S_v_O_2_* is the mixed venous oxygen saturation, available in the simulation model as the flow-weighted mean value of oxygen saturation in all blood returning from the systemic capillary beds, but differs substantially from both the pre-oxygenator saturation and pulmonary arterial saturation ^1,4,8^.

**Calculations**

The program version used was Aplysia CardioVascular Lab 6.6.0.3 (Aplysia Medical AB, Stockholm, Sweden). Mean values in the model were calculated as running arithmetic means. All data were collected at end-diastole after at least 2 minutes simulation to allow for steady-state conditions regarding hemodynamics and oxygen transport. **Pressures, flows, volumes and saturations in every compartment were updated with 4000 Hz.** The cardiovascular simulation results; mean arterial pressure, cardiac output, systemic blood flow, stroke work, LV oxygen consumption and coronary flow in all scenarios are presented in Table 1 and Figure 1-9. Results are commented and interpreted in the main text.

**ECMO simulation**

VA ECMO was simulated with a fixed blood flow (0-4 L/min) mimicking a peripheral, bi-femoral cannulation with right atrial venous drainage and retrograde reinfusion in the descending aorta (Figure 2). Since ECMO flow was constant neither elastic nor inertial properties of the cannulae and tubings were included in the simulation. The fixed blood flow can be seen as originating from a roller pump, although the small pulsatility seen in the real world due to the rotating rollers are not included in the model.

**Simulation of inotropic support**

The myocardial reserve recruitable with pharmacological inotropic support in cardiogenic shock is difficult to predict ^9^. We have simulated inotropic support by increasing left ventricular end-systolic elastance from 0.5 to 0.8 mmHg/mL, while adjusting MAP to 65 mmHg, mimicking the clinical effect of inodilator therapy (without changing heart rate). This results in LV ejection fraction increasing from 12% to 26% and stroke volumes from 18 to 36 mL.

**Simulation of Intra-Aortic Balloon Pump**

The physiological effect of the aortic balloon pump was mimicked by diastolic displacement of 30 mL of blood from the descending aortic compartment, with only partial occlusion of flow in the vascular segment allowing perfusion from the ECMO cannula of vascular beds both above and below the balloon. The net effect is both a decrease in afterload with a modest increase in LV output and an augmentation of aortic diastolic pressure and coronary perfusion. The flow resistance of the descending aorta is dynamically updated depending on the balloon volume, but balloon size is set not to occlude the aorta even if fully inflated.

**Simulation of Impella®**

The axial pump is placed through the aortic valve in the model without any aortic regurgitation and flow is set between 1.0 L/min and 5.0 L/min to mimic commercially available devices (Impella® 2.5, CP and 5.0). The net effect is a decrease in both LV preload and afterload, but without a selective augmentation of diastolic coronary perfusion as with the intra-aortic balloon pump. The Impella® is simulated as a centrifugal pump with a pump head described by the equation below ^10^.

$$h = \frac{(p2 - p1)}{\rho\cdot g}+\frac{v^{2}}{2\cdot g}$$

h = total head developed (m)
p_2_ = pressure at outlet (ascending aorta) (N/m^2^)
p_1_ = pressure at inlet (left ventricle) (N/m^2^)

ρ =  blood density (kg/m^3^)
g = acceleration of gravity (9.81) m/s^2^v = velocity at the outlet (m/s)

Outlet velocity *v* is determined by the rotational speed (up to 40.000 rpm) and radius (0.2 cm) of the pumphead. Pump flow is influenced both by the actual left ventricular pressure and the aortic pressure, therefore some pulsatility may be preserved despite a closed aortic valve.

**Simulation of Atrial Septal Defects**

Atrial septal defect flow is simulated as flow through a valve with a fixed diameter ^2^. A virtual length is assigned to the defect, set at a value identical to the diameter of the defect. This length is used to calculate the flow inertia in the inflow region of the defect. The pressure gradient through a valve or stenosis can be calculated with one resistance term proportional to flow, one proportional to squared flow and one inertia term proportional to the time derivative of flow as described by Stergiopulos *et al ^11^*.

$$\Delta p_{s}=\frac{4\cdot K_{v}\cdot\mu}{\pi\cdot D_{0}^{3}}\cdot q_{s}+\frac{\rho\cdot K_{t}}{2\cdot A_{0}^{2}}\cdot\left( \frac{A_{0}}{A_{s}}-1 \right)^{2}\cdot q_{s}^{2}+\frac{\rho\cdot K_{u}\cdot l_{s}}{A_{0}}\cdot\frac{dq_{s}}{dt}$$

$\Delta$p_s_ Stenosis pressure gradient
K_v_ Dimensionless empirical constant
μ B viscosity (g/cm/s)

- Mathematical constant (3.1416)

D_0_ Diameter before/after stenosis

q_s_ Stenosis flow
ρ Blood density (1.06 g/mL)

K_t_ Dimensionless empirical constant (1.52)
A_0_ Area before/after stenosis
A_s_ Area of stenosis (vena contracta)
K_u_ Dimensionless empirical constant (1.20)
l_s_ Effective length of stenosis

This complex semi-empirical equation may be simplified to the equation shown below if A_0_ >>A_s_ with similar results as is used for valves and shunts in this and many other cardiovascular simulation models ^3,12^.

$$\Delta p_{s}=B\cdot q_{s}\cdot\left| q_{s} \right|+L\cdot\frac{dq_{s}}{dt}$$

$$B=\frac{\rho}{2\cdot A_{eff}}$$

$$L=\frac{\rho\cdot l_{eff}}{A_{eff}}$$

A_eff_ is the effective area of the valve, stenosis or shunt. l_eff_ is the effective length, which is assumed to be identical to the diameter of the effective area A_eff_ (Personal communication Theo Arts, Maastricht).

**Simulation of venting**

Venting in the model is passive with flow proportional to the pressure gradient between the vented compartment (pulmonary artery, left atrium or left ventricle) and the resistance of two meters long 3/8” tubing with laminar flow. Flow is uni-directional from the vented compartment to the venous side of the ECMO circuit.

**Patient-specific simulation**

The large number of model parameters cannot unequivocally be determined by available clinical data alone. Many of the parameters are however based on known anatomical and tissue properties and can therefore be considered as generic based on the size and age of the patient. A sensitivity analysis has shown that parameters related to filling and ventricular stiffness are the most important in determination of cardiac output, while arterial stiffness and arteriolar vascular tone is important for vascular pressures ^2^. Systolic contractility is the major determinant of ventricular ejection fraction. Blood volume and the mentioned model parameters are adjusted in a recursive way until the best fit with clinical data appears.

## Supplemental Videos. Animated simulations of cardiogenic shock, VA ECMO and LV unloading interventions

1. Creation of systolic left heart failure with decrease in left heart contractility and increase in LV stiffness and finally blood volume increase of 800 mL to mimic secondary fluid retention.
2. Cardiogenic shock with VA ECMO flows 0-4 L/min. An increase in left ventricular loading is seen.
3. Cardiogenic shock with VA ECMO flows 0-4 L/min. First decrease of blood volume by 800 mL and the decrease of systemic vascular resistance to mean arterial pressure 65 mmHg. A decrease in blood volume and systemic vascular resistance are reasonable first order unloading measures.
4. Cardiogenic shock with VA ECMO flows 0-4 L/min. Start and stop of intra-aortic balloon pumping (IABP). The unloading effect of IABP is limited, although an increase in coronary blood flow and conversion of a non-ejecting left ventricle to an ejecting ventricle in extreme cases may justify its use.
5. Cardiogenic shock with VA ECMO flows 0-4 L/min. Start of Impella 0-5 L/min. Finally decrease of systemic vascular resistance to mean arterial pressure 65 mmHg. Unloading of the left ventricle is good, although ejected left ventricular blood may be hypoxic in extreme cases of pulmonary congestion.
6. Cardiogenic shock with VA ECMO flows 0-4 L/min. Atrial septal defects 0-0.5-1.0-1.5 cm2. Finally increase of systemic vascular resistance to mean arterial pressure 65 mmHg. Unloading of the left ventricle is good, although a risk of a non-ejecting left ventricle with thrombus formation exists.

Animated tutorials 7-12 provide more explanatory comments and show the full simulation software interface corresponding to pressure-volume loop animations 1-6:

1. Simulation of left ventricular heart failure (PV loops in animation 1)
2. Start of VA ECMO in cardiogenic shock due to left heart failure (PV loops in animation 2)
3. Cardiogenic shock with VA ECMO flow 4 L/min. Decrease in afterload and blood volume (PV loops in animation 3).
4. Cardiogenic shock with VA ECMO flow 4 L/min. Effects of intraaortic balloon pump. (PV loops in animation 4).
5. Cardiogenic shock with VA ECMO flow 4 L/min. Effects of Impella 0-5 L/min. (PV loops in animation 5).
6. Cardiogenic shock with VA ECMO flow 4 L/min. Effects of atrial septostomy (PV loops in animation 6).

**Figures**

**Figure A1**


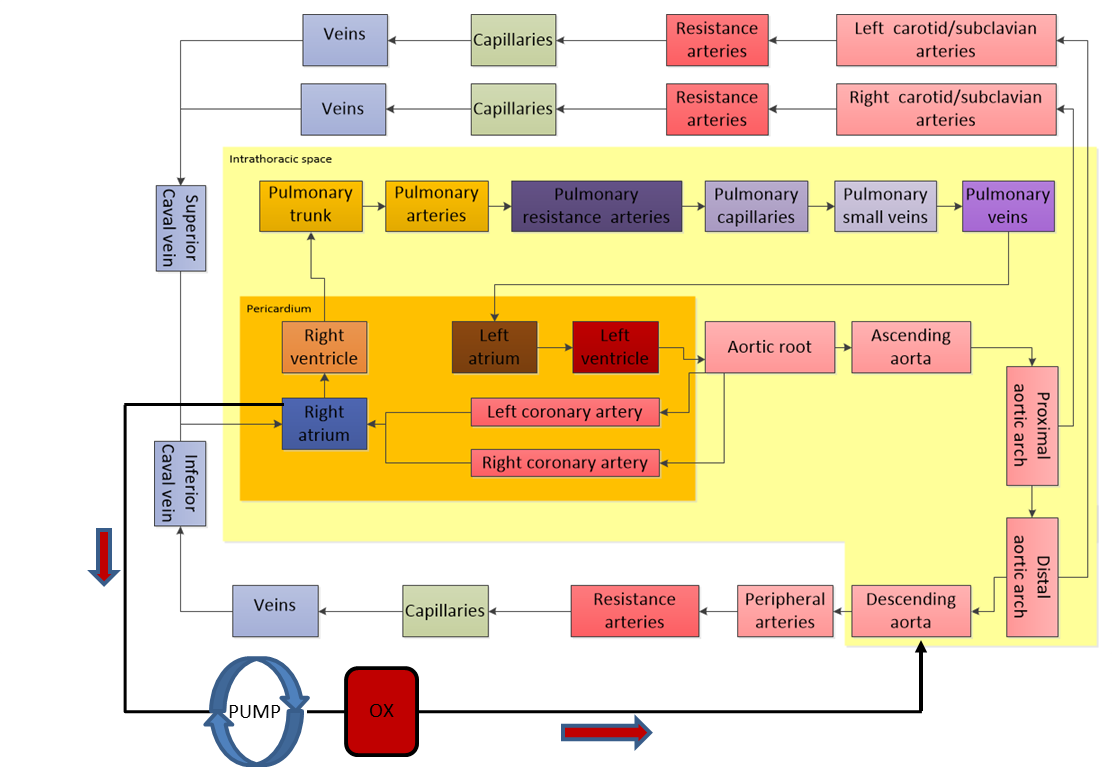


**Figure A2**


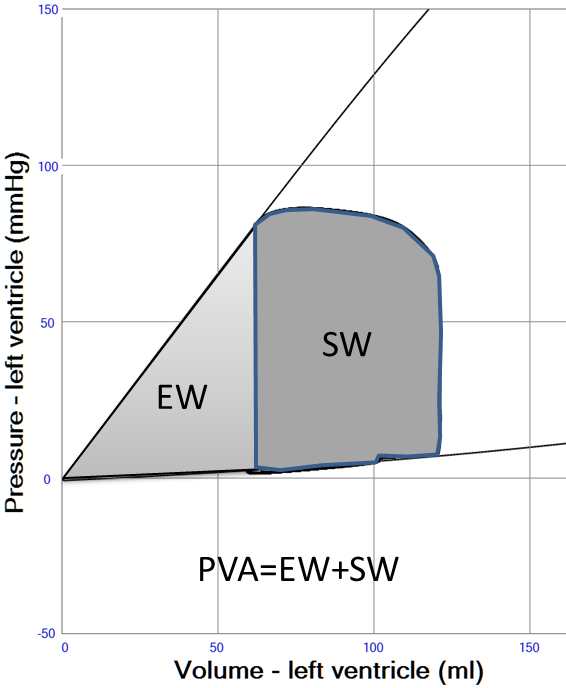


**Figure A3**


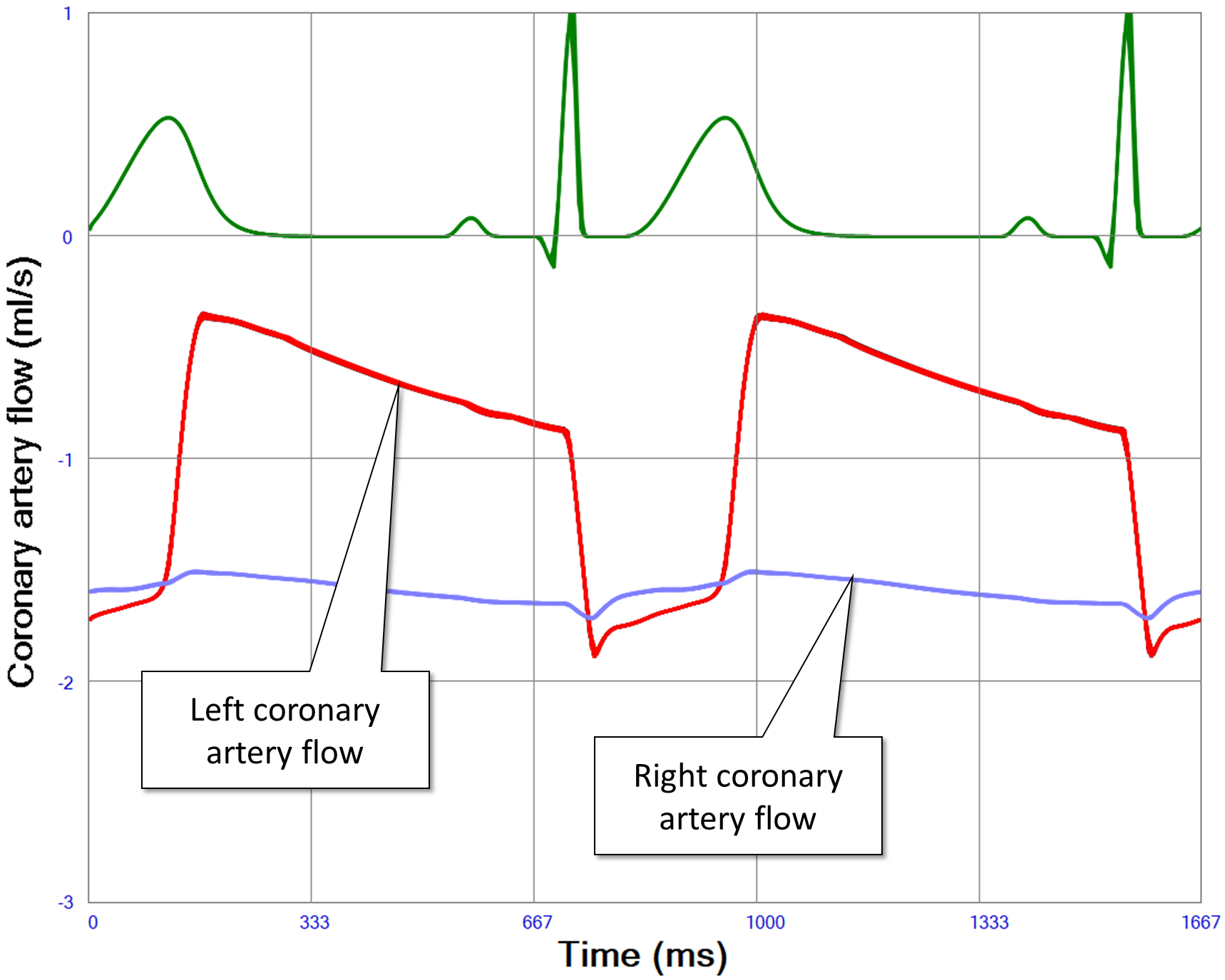


#

# Figure legends

**Figure A1.** Block diagram with an overview of the simulation model. The connections of a peripheral veno-arterial ECMO system through the femoral vessels is shown in the lower left corner. The arrows indicate blood flow direction, OX membrane oxygenator and PUMP centrifugal blood pump.

**Figure A2.** Pressure-Volume Area (PVA) is the total work of the left ventricle during one cardiac cycle. It is calculated as the sum of stroke work (SW) and elastic work (EW). PVA is linearly related to left ventricular oxygen consumption according to Suga et al ^5^.

**Figure A3.** Coronary flow supplying the left and right side of the heart is shown in a simulation of a case exhibiting normal physiology, indicating that flow curves are realistic when determining flow with a fixed resistance and a variable driving pressure calculated as the difference between aortic root pressure and estimated wall tension. The LV is mainly perfused during diastole, while the right ventricle is perfused throughout the entire cardiac cycle.

# References

1. Broman M, Frenckner B, Bjallmark A, Broome M: Recirculation during veno-venous extra-corporeal membrane oxygenation--a simulation study. *Int J Artif Organs* 38 (1): 23-30, 2015 doi: 10.5301/ijao.5000373.

2. Broome M, Donker DW: Individualized real-time clinical decision support to monitor cardiac loading during venoarterial ECMO. *Journal of translational medicine* 14 (1): 4, 2016 doi: 10.1186/s12967-015-0760-1.

3. Broome M, Maksuti E, Bjallmark A, Frenckner B, Janerot-Sjoberg B: Closed-loop real-time simulation model of hemodynamics and oxygen transport in the cardiovascular system. *Biomed Eng Online* [Research Support, Non-U.S. Gov't] 12: 69, 2013 doi: 10.1186/1475-925X-12-69.

4. Lindfors M, Frenckner B, Sartipy U, Bjallmark A, Broome M: Venous Cannula Positioning in Arterial Deoxygenation During Veno-Arterial Extracorporeal Membrane Oxygenation-A Simulation Study and Case Report. *Artif Organs* 41 (1): 75-81, 2017 doi: 10.1111/aor.12700.

5. Suga H: Total mechanical energy of a ventricle model and cardiac oxygen consumption. *Am J Physiol* 236 (3): H498-505, 1979.

6. Takaoka H, Takeuchi M, Odake M, Yokoyama M: Assessment of myocardial oxygen consumption (Vo2) and systolic pressure-volume area (PVA) in human hearts. *Eur Heart J* 13 Suppl E: 85-90, 1992.

7. Hüfner CG: Neue Versuche zur Bestimmung der Sauerstofcapacität. *Arch Physiol* 12: 130-176, 1902.

8. Hou X, Yang X, Du Z*, et al*: Superior vena cava drainage improves upper body oxygenation during veno-arterial extracorporeal membrane oxygenation in sheep. *Crit Care* 19: 68, 2015 doi: 10.1186/s13054-015-0791-2.

9. Reynolds HR, Hochman JS: Cardiogenic shock: current concepts and improving outcomes. *Circulation* 117 (5): 686-97, 2008 doi: 10.1161/CIRCULATIONAHA.106.613596.

10. Unknown: An introduction to Centrifugal Pumps. Available at: https://www.engineeringtoolbox.com/centrifugal-pumps-d_54.html. Accessed 8th of November 2017, 2017.

11. Stergiopulos N, Spiridon M, Pythoud F, Meister JJ: On the wave transmission and reflection properties of stenoses. *J Biomech* 29 (1): 31-8, 1996.

12. Mynard JP, Davidson MR, Penny DJ, Smolich JJ: A simple versatile model of valve dynamics for use in lumped parameter and one-dimensional cardiovascular models. *International Journal for Numerical Methods in Biomedical Engineering* 28 (6-7): 626-641, 2011.
